# Burnout in the staff of a chronic care hospital

**DOI:** 10.11606/S1518-8787.2018052000242

**Published:** 2018-04-20

**Authors:** Maria Jose Merino-Plaza, Francisco Javier Carrera-Hueso, Nuria Arribas-Boscá, Amparo Martínez-Asensi, Emilia Trull-Maravilla, Narjis Fikri-Benbrahim

**Affiliations:** I Hospital Doctor Moliner. Serra. Valencia, España; II Universidad de Granada. Granada, España; III Unidad de Salud Mental de Foios. Hospital Clínico. Valencia, España

**Keywords:** Burnout, Professional, classification, Patient Care Team, Hospitals, Public, manpower, Working Conditions, Socioeconomic Factors, Agotamiento Profesional, clasificación, Grupo de Atención al Paciente, Hospitales Públicos, Recursos Humanos, Condiciones de Trabajo, Factores Socioeconómicos

## Abstract

**OBJECTIVE:**

To estimate the prevalence of Burnout in a medium or long-stay hospital, to monitor its evolution and to highlight the importance of cut-off points used to avoid distortions in the interpretation of the results.

**METHODS:**

Two cross-sectional studies (2013–2016) were carried out, applying the Spanish version of the Maslach Burnout Inventory to the staff of a chronic care hospital (n = 323). Result variables were: Burnout prevalence and a high degree of affectation of the subscales and predictor variables: sociodemographic characteristics and factors that trigger and modulate the syndrome. The association between variables was quantified using odds ratio.

**RESULTS:**

The participation rate went from 31.5% to 39.3%. The professionals presented a mean level of Burnout in both moments, observing a lower degree of affectation of the depersonalization subscales and personal accomplishment in the 2016 cut-off. The average score of the subscales in 2016 was 21.5 for emotional fatigue, 4.7 for depersonalization and 41.7 for personal fulfillment, compared to the values of emotional fatigue = 21.6, depersonalization = 6.9 and personal fulfillment = 36.3 obtained in 2013. The emotional fatigue score was slightly higher than the mean value of the national studies (19.9), while the rest of the values were similar to the mean values of the studies considered. The prevalence of Burnout and the interpretation of the results varied significantly according to the cut-off points considered. In both studies, sociodemographic variables showed little significance, while social support and interpersonal relationships were associated with the degree of burnout among professionals.

**CONCLUSIONS:**

Our prevalence of Burnout was similar to that of other studies consulted, although the emotional component is more marked in our environment. The interpretation of the results varied significantly according to the cut-off points applied, due to the cross-cultural differences.

## RESUMEN

**OBJETIVO:**

Estimar la prevalencia de Burnout en un hospital de media-larga estancia, monitorizar su evolución y evidenciar la importancia de los puntos de corte utilizados para evitar sesgos en la interpretación de los resultados.

**MÉTODOS:**

Se realizaron dos estudios transversales (2013–2016), aplicando la versión española del cuestionario Maslach Burnout *Inventory* al personal de un hospital de crónicos (n = 323). Fueron variables resultado: prevalencia de Burnout y alto grado de afectación de las subescalas y variables predictoras: características sociodemográficas y factores desencadenantes y moduladores del síndrome. La asociación entre variables se cuantificó mediante *odds ratio*.

**RESULTADOS:**

El índice de participación pasó del 31,5% al 39,3%. Los profesionales presentaron un nivel medio de Burnout en ambos momentos, observándose menor grado de afectación de las subescalas de despersonalización y realización personal en el corte realizado en 2016. La puntuación media de las subescalas en 2016 fue 21,5 para el cansancio emocional, 4,7 para la despersonalización y 41,7 para la realización personal, frente a los valores de cansancio emocional = 21,6, despersonalización = 6,9 y realización personal = 36,3 obtenidos en 2013. La puntuación de la escala de cansancio emocional fue ligeramente superior al valor promedio de los estudios nacionales (19,9), mientras que el resto de valores fueron similares a los valores promedio de los estudios considerados. La prevalencia de Burnout y la interpretación de los resultados variaron significativamente en función de los puntos de corte considerados. En ambos estudios, las variables sociodemográficas mostraron escasa significación, mientras que el apoyo social y las relaciones interpersonales se asociaron al grado de Burnout de los profesionales.

**CONCLUSIONES:**

Nuestra prevalencia de Burnout fue similar a la de otros estudios consultados, aunque el componente emocional es más marcado en nuestro medio. La interpretación de los resultados varió significativamente en función de los puntos de corte aplicados, debido a las diferencias transculturales.

## INTRODUCTION

Burnout or Occupational Burnout appears due to an inadequate response to chronic work stressors of an interpersonal nature, with negative consequences for personal, work and organizational levels^1^. It mainly affects professionals who provide their services in direct contact with the recipient of their care, such as health personnel, teachers or police. Its origin is occupational, but its etiology is multifactorial and depends on individual, social and organizational factors.

The Maslach Burnout Inventory (MBI)^2^ is the most commonly used instrument for its evaluation and contributes to systematizing the research on the construct. Their subscales were not identified from a theoretical model supported by clinical observation but were deduced from exploratory studies carried out to confirm the factorial validity of the tool^3^ ^,^ ^4^.

Christina Maslach considers Burnout a three-dimensional syndrome, although some authors propose a two-dimensional structure and others expose a factorial structure of four or more dimensions^5^. However, all consider emotional fatigue as the main nucleus of the syndrome, and it is the most stable and predictive component of its consequences^1^ ^,^ ^6^. The disparity in results on the factor structure and psychometric properties of MBI is due to the heterogeneity of the samples used in the different studies. There is no unanimous criterion on the cut-off points to be used to diagnose burnout and to calculate its prevalence, but the instrument has been adapted to different languages to avoid distortions in the interpretation of the results^5^ ^,^ ^7^ ^,^ ^8^. Gil-Monte and Peiró validated the factorial structure of MBI for the Spanish population in 1999 and established cut-off points for this population in 2000^9^.

The health environment supports, among others, three characteristics that are risk factors for the appearance of Burnout: a great mental and physical effort, continuous interruptions and reorganization of tasks, which increase the mental load and effort and dealing with the patient in complex circumstances of anxiety and pain. In addition, the health system is increasingly complex and subject to organizational and technological changes that cause professionals to adapt continuously, increasing stress in the perceived work and the risk of the onset of the syndrome.

Several studies on Burnout were carried out in primary care, specialized hospitals, chronic diseases hospitals and specific areas and services at a national and international level^6^ ^,^ ^10^. However, there are no surveys conducted in medium-long-term hospitals, where, because of the great vulnerability of their patients, it can produce more emotional involvement of the staff than in other types of health institutions.

The objective of the present study was to estimate the prevalence of Burnout in a medium-long stay Hospital, to monitor its evolution and to highlight the importance of the cut-off points used to avoid distortions in the interpretation of results.

## METHODS

The study population was the staff of the Doctor Moliner hospital, a public, medium-long stay hospital with 183 beds located in the province of Valencia, Spain. Two serial observational studies were conducted in March 2013 and 2016 to determine the prevalence of Burnout. Participation was voluntary and anonymous.

The instrument used was the Spanish version of the second edition of the Maslach Burnout Inventory-Human Services Survey (MBI-HSS)^11^ aimed at health professionals.

The questionnaire applied had three parts. The first one collected sociodemographic characteristics of the respondents. The second asked for information on triggering factors and modulators described in the literature^1^. The triggering factors evaluated were: interpersonal relationships between professionals and patients (self-perception of the value of family members and patients), interpersonal relationships at work (self-perception of peers and bosses), and professional appreciation of perceived gains. The modulating factors evaluated were: training, social support, the perception of one's professional experience and certain personal variables, such as self-efficacy and optimism. The training and interpersonal relationships (valuation as patients, relatives, partners and superiors), were evaluated with a dichotomous scale (yes, no). Professional experience and perceptions about self-efficacy, optimism, gains and social support were assessed on a discrete scale from one to 10. The third part of the questionnaire included the 22 questions from the MBI-HSS test that assessed the degree of affectation of the subscales that define the syndrome – emotional fatigue (EF), depersonalization (DP) and low personal fulfillment (PF). These questions were answered on a seven-degree Likert scale ranging from zero (never) to six (every day).

The questionnaires were considered valid if all MBI-HSS test questions were answered, although there was some lack of response to the variables collected in the first and second part of the questionnaire.

In order to categorize professionals in individuals with high, medium and low affectation levels of the subscales in 2013, the 33rd and 66th percentiles were calculated according to the criteria proposed by Maslach and Jackson^2^ and were used as their own cut-off points in both the moments. In addition, to verify if there were differences or distortions in the interpretation of the results according to the cut-off points applied, the cut-off points described in five reference studies regarding Burnout among health professionals were used^2^ ^,^ ^9^ ^,^ ^11^ ^–^ ^13^.

The sample size was not predetermined since the questionnaires were sent by internal mail to all staff in both cut-offs. The procedure used for sampling and data collection was the same in both studies. At both times, a letter was sent alongside the research, explaining the objectives of the study and asking for the collaboration of the professionals. The questionnaire was self-administered, was distributed in February and responses were collected in March through the suggestion boxes to ensure anonymity. The group had 323 employees in 2013; 318 acknowledgments of receipt were received. The remaining five did not receive the survey for various reasons ((free days vacations, etc.). A total of 125 responses were obtained (39.3% of the participants), of which 100 were valid (participation index = 31.5%). By 2016, the group had 312 employees but received only 257 acknowledgments of receipt. A total of 121 responses (47.1% of participants) were obtained, of which 101 were valid (participation rate = 39.3%).

Position (mean) and dispersion (standard deviation) measures were used to describe the quantitative variables and frequency measures (percentages) for the categorical variables. The independent variables were the sociodemographic characteristics of the staff and their perception of the triggering factors and modulators evaluated. The variables were the degree of affectation of the subscales (score ≥ to the cut-off points considered) and the presence of Burnout (high degree of affectation of the three subscales).

To verify the normal distribution of variables, the Shapiro-Wilk test was used, using parametric models if they were met or their non-parametric equivalents otherwise. In the bivariate analysis, the chi-square test was used to relate qualitative variables, the t-Student test for quantitative variables and the one-way ANOVA test for quantitative variables against qualitative variables of three or more categories, with the Bonferroni post hoc correction.

The average scores of the subscales were compared with those obtained in seven similar studies carried out in Spain in the last few years^7^ ^,^ ^10^ ^,^ ^14^ ^-^ ^18^.

To transform the variables evaluated by discrete scale into categorical variables, a new variable called “high perception” was created, considering that this level was reached when its score was higher than eight. For the Burnout subscales, a new variable named ‘high affectation’ was coded, considering that this level was reached when the Gil-Monte cut-off points were exceeded^9^. The odds ratio (OR) and its 95% confidence interval (CI) were used to quantify the degree of association of the independent variables with the Burnout subscales. The adjusted analysis was performed using binary logistic regression, including in the model the potentially confounding variables according to the Maldonado-Greeland criterion^19^.

Statistical analysis was performed using the IBM SPSS Statistics program, version 19.0.

The study was approved by the Management and the Commissions of Quality, Bioethics, and Teaching and Research. For the application of the instrument, the explicit consent of the participants was not requested, since the participation was voluntary and anonymous, the response and delivery of the completed research implied that consent and was included in the prior information given to the staff.

## RESULTS

The sociodemographic data of the group and the characteristics of the professionals participating in the research were presented in Table 1. Significant differences were observed in relation to the professional category and the time worked of the participants in both moments of the study, with an increase in the participation of the group of doctors and professionals with more than 20 years working. Regarding the triggering factors and modulators, we observed a better self-perception of the valorization by the relatives in the 2016 cut-off.

**Table 1:** Sociodemographic characteristics of the Hospital group and of the research participants (2013–2016).

2013 Group	2016 Group	Population characteristics of survey participants		2013	2016	Statistical significance
Mean (SD)/n (%)	Mean (SD)/n (%)			(n = 100)^a^	(n = 101)^a^	p (95%CI)
Age: 44.25 (8.2)	Age: 48.4 (8.7)	Age (years) - mean (SD)		45.1 (8.96)	47.2 (9.68)	p = 0.50
Female: 262 (81.2)	Female: 251 (80.4)	Gender - n (%)				p = 0.71
Type of contract	Type of contract		Women	80 (81.6)	82 (83.7)	
Fixed: 78 (24.2)	Fixed: 80 (25.6)	Type of contract - n (%)				p = 0.99^a^
Non-fixed: 245 (75.8)	Non-fixed: 232 (74.4)		Fixed:	38 (38.0)	41 (41.4)	
Professional category	Professional category		Interim	53 (53.0)	48 (48.5)	OR = 0.84 (0.47–1.51)
Doctors: 25 (7.7)	Doctors: 25 (8)		Accumulation of tasks	1 (1.0)	0 (0.0)	Not available for evaluation
NUG: 96 (29.7)	NUG: 92 (29.5)		Eventual	8 (8.0)	10 (10.1)	OR = 1.16 (0.41–3.24)
Nursing Assistant: 91 (28.2)	Nursing assistant: 89 (28.5)	Professional category - n (%)				**p = 0.02** a.^b^
Janitors: 42 (13)	Janitors: 41 (13.1)		Doctors	6 (6.1)	16 (16.0)	
Others 69 (21.4)	Others 65 (20.7)		NUG	39 (39.4)	44 (44.0)	OR = 0.42 (0.15–1.19)
			Nursing assistant	37 (37.4)	33 (33.0)	OR = 0.33 (0.12–0.95)
			Janitors	11 (11.1)	5 (5.9)	OR = 0.17 (0.04–0.70)
			Others	6 (6.1)	2 (2.0)	OR = 0.13 (0.02–0.80)
		Years of experience - n (%):				**p = 0.03** a.^b^
			< 5	8 (8.0)	4 (4.0)	
			5–9	20 (20.0)	17 (17.0)	OR = 1.70 (0.44–6.65)
			10–14	26 (26.0)	19 (19.0)	OR = 1.46 (0.38–5.57)
			15–19	21 (21.0)	21 (21.0)	OR = 2.00 (0.52–7.67)
			≥ 20	25 (25.0)	39 (39.0)	OR = 3.12 (0.85–11.4)
		Years of experience - n (%):				p = 0.90^a^
			< 5	42 (42.4)	40 (39.6)	
			5–9	29 (29.3)	30 (29.7)	OR = 1.09 (0.56–2.12)
			10–14	18 (18.2)	8 (7.9)	OR = 0.47 (0.18–1.19)
			15–19	5 (5.1)	7 (6.9)	OR = 1.47 (0.43–5.01)
			≥ 20	5 (5.1)	7 (6.9)	OR = 1.47 (0.43–5.01)
		Marital status - n (%):				p = 0.43^a^
			Single	19 (19.0)	14 (14.6)	
			Married or with family	66 (66.0)	67 (69.8)	OR = 1.38 (0.64–2.97)
			Divorced	12 (12.0)	12 (12.5)	OR = 1.36 (0.47–3.90)
			Widowed	2 (2.0)	3 (3.1)	OR = 2.04 (0.30–13.85)
		Firings in the last year - n (%)		23 (23.0)	17 (16.8)	p = 0.27
		Chronic disease - n (%)		30 (30.6)	25 (24.8)	p = 0.36
		Received specific training - n (%)		71 (72.4)	61 (61.0)	p = 0.09
		Feels valued by patients - n (%)		71 (83.5)	80 (92.0)	p = 0.09
		Feels appreciated by family members - n (%)		62 (73.8)	72 (86.7)	**p = 0.04** b
		Feels appreciated by co-workers - n (%)		78 (85.7)	78 (84.8)	p = 0.86
		Feels appreciated by bosses - n (%)		59 (66.3)	64 (68.8)	p = 0.72
		What is your overall appreciation of your work experience? - mean (SD)		7.73 (1.78)	7.55 (1.72)	p = 0.48
		Do you consider yourself an optimist? - mean (SD)		7.79 (1.91)	7.83 (1.65)	p = 0.87
		What is the value of your earnings in relation to the work you do? - mean (SD)		4.79 (2.51)	4.89 (2.29)	p = 0.77
		Do you believe that you perform well in your daily work (self-efficacy)? - mean (SD)		8.43 (1.41)	8.37 (1.16)	p = 0.74
		Do you feel supported in your personal life (friends, family etc.)? - mean (SD)		8.55 (1.65)	8.90 (1.17)	p = 0.09

SD: standard deviation; NUG: nursing university graduate; OR: odds ratio

a Mantel-Hanzel test.

b p < 0.05

The reference category is the first.

Values with statistical significance are highlighted in bold.

The mean scores of the subscales at both moments and their 33 and 66 percentiles were presented in Table 2. The values obtained place our professionals in an average level of professional fatigue, although the fatigue observed in 2016 was lower, with significant differences between the two moments for DP and PF.

**Table 2:** Mean scores and 33 and 66 percentiles of the subscales obtained in the study, 2013–2016.

Subscales		2013	2016	Difference (95%CI)*	Statistical significance p*
Emotional fatigue	Mean (SD)	21.6 (10.7)	21.5 (12.9)	-0.07 (-3.37–3.23)	-
	33 percentile	17	14	-3.0 (-0.3–6.3)	-
	66 percentile	25	26	1 (-2.3–4.3)	-
Depersonalization	Mean (SD)	6.9 (5.2)	4.7 (5.2)	-2.20 (-3.65– -0.76)	**0.003**
	33 percentile	4	4	0 (-1.44–1.44)	-
	66 percentile	8	6	-2 (0.56–3.44)	< **0.05**
Personal fulfillment	Mean (SD)	36.3 (8.0)	41.7 (6.8)	5.34 (3.27–7.40)	< **0.001**
	33 percentile	38	46	8 (5.94–10.1)	< **0.05**
	66 percentile	34	40	6 (3.94–8.06)	< **0.05**

SD: Standard deviation

* Differences = 2016 minus 2013; (t-Student).

Values with statistical significance are highlighted in bold.

Our average for the EF in the 2016 cut-off (21.5) was higher than the mean value of the seven studies consulted (19.9). Our mean for the PF scale was also higher than the mean value of these studies (PF_2016_ = 41.7 *versus* 34.4), while our score on the DP scale was lower than the mean value (DP_2016_ = 4.7 *versus* 7.1).

The results of Burnout prevalence and degree of affectation of the subscales varied greatly according to the normative criteria applied. In Table 3, the results obtained in both moments were presented, comparing their interpretation according to the normative values considered. The most restrictive criterion was that of Schaufeli.

**Table 3:** Burnout frequency and degree of affectation of the subscales (MBI-HSS) obtained in the study according to the cut-off points proposed by different authors (2013–2016).

Normative criteria	Subscales	Cut-off points	Degree of affectation	2013	2016	Significación estadística p*
	Mean (SD)			n (%)	n (%)	
Cut-off points per tertiles, as recommended by Maslach Cut made in 2013 (n = 100)	Emotional fatigue 21.6 (10.7)	≤ 17	Low	37 (37.0)	46 (45.5)	0.69
		18–25	Medium	31 (31.0)	20 (19.8)	
		> 25	High	32 (32.0)	35 (34.7)	
	Depersonalization 6.9 (5.2)	≤ 4	Low	33 (33.0)	57 (56.4)	**0.007**
		5–8	Medium	35 (35.0)	28 (27.7)	
		> 8	High	32 (32.0)	16 (15.8)	
	Personal fulfillment 36.3 (8.0)	> 38	Low	34 (34.0)	75 (74.2)	**< 0.001**
		34–38	Medium	33 (33.0)	14 (13.9)	
		≤ 34	High	33 (33.0)	12 (11.9)	
	Burnout	n (%)		5 (5.0)	3 (3.0)	0.46
Cut-off points of Maslach and Jackson EEUU, 1986 (health personnel) (n = 11,067)	Emotional fatigue 21.0 (10.7)	< 17	Low	32 (32.0)	41 (40.6)	0.24
		17–26	Medium	41 (41.0)	25 (24.8)	
		≥ 27	High	27 (27.0)	35 (34.7)	
	Depersonalization 8.7 (5.9)	< 7	Low	49 (49.0)	76 (75.2)	0.62
		7–12	Medium	40 (40.0)	16 (15.8)	
		≥ 13	High	11 (11.0)	9 (8.9)	
	Personal fulfillment 34.6 (7.1)	≥ 39	Low	34 (34.0)	75 (74.3)	**0.01**
		32–38	Medium	45 (45.0)	20 (19.8)	
		< 32	High	21 (21.0)	6 (5.9)	
	Burnout	n (%)		3 (3.0)	1 (1.0)	0.31
Cut-off points of Seisdedos Spain, 1997 (multi-occupational sample) (n = 1,138)	Emotional fatigue 22.2 (9.3)	< 15	Low	25 (25.0)	36 (35.6)	0.60
		15–24	Medium	39 (39.0)	25 (24.8)	
		≥ 25	High	36 (36.0)	40 (39.6)	
	Depersonalization 7.2 (5.2)	< 4	Low	27 (27.0)	56 (55.4)	0.07
		4–9	Medium	47 (47.0)	29 (28.7)	
		≥ 10	High	26 (26.0)	16 (15.8)	
	Personal fulfillment 36.5 (7.3)	≥ 40	Low	29 (29.0)	70 (69.3)	**0.002**
		33–39	Medium	49 (49.0)	24 (23.8)	
		< 33	High	22 (22.0)	7 (6.9)	
	Burnout	n (%)		7 7.0)	3 (3.0)	0.19
Cut-off points of Gil-monte and Peiró Spain, 2000 (multi-occupational sample) (n = 1,188)	Emotional fatigue 20.4 (11.0)	< 16	Low	30 (30.0)	39 (38.6)	0.60
		16–24	Medium	34 (34.0)	22 (21.8)	
		≥ 25	High	36 (36.0)	40 (39.6)	
	Depersonalization 6.4 (5.3)	< 4	Low	27 (27.0)	56 (55.4)	**0.01**
		4–8	Medium	41 (41.0)	28 (27.7)	
		≥ 9	High	32 (32.0)	17 (16.8)	
	Personal fulfillment 36.0 (7.3)	≥ 40	Low	29 (29.0)	70 (69.3)	**< 0.001**
		36–39	Medium	30 (30.0)	15 (14.9)	
		< 36	High	41 (41.0)	16 (15.8)	
	Burnout	n (%)		14 (14.0)	5 (5.0)	**0.03**
Cut-off points of Neira Argentina, 2004 (health personnel) (n = 1,152)	Emotional fatigue 18.7 (11.4)	< 12	Low	17 (17.0)	23 (22.8)	0.61
		12–21	Medium	42 (44.0)	33 (32.7)	
		≥ 22	High	41 (41.0)	45 (44.6)	
	Depersonalization 6.1 (5.8)	< 3	Low	20 (20.0)	45 (44.6)	**< 0.001**
		3–6	Medium	29 (29.0)	31 (30.7)	
		≥ 7	High	51 (51.0)	25 (24.8)	
	Personal fulfillment 37.3 (7.8)	≥ 41	Low	29 (29.0)	70 (69.3)	**< 0.001**
		36–40	Medium	30 (30.0)	15 (14.9)	
		< 36	High	41 (41.0)	16 (15.8)	
	Burnout	n (%)		20 (20.0)	5 (5.0)	**0.001**
Cut-off points of Schaufeli Holanda, 1995 (multi-occupational sample diagnosed with Burnout) (n = 142)	Emotional fatigue 28.6 (10.1)	<26	Low	17 (17.0)	23 (22.8)	0.61
		26-33	Medium	42 (42.0)	33 (32.7)	
		≥ 34	High	41 (41.0)	45 (44.6)	
	Depersonalization 9.3 (4.9)	< 6	Low	40 (40.0)	66 (65.3)	0.15
		6-11	Medium	42 (42.0)	24 (23.8)	
		≥ 12	High	18 (18.0)	11 (10.9)	
	Personal fulfillment 27.0 (5.7)	≥ 29	Low	86 (86.0)	98 (97.0)	0.11
		26-28	Medium	6 (6.0)	0 (0.0)	
		< 26	High	8 (8.0)	3 (3.0)	
	Burnout	n (%)		3 (3.0)	1 (1.0)	0.31*

SD: standard deviation; MBI-HSS: Maslach Burnout Inventory

* Differences in the high degree of affectation of the subscales and the prevalence of Burnout (2016 *versus* 2013) (Chi-square test).

Values with statistical significance are highlighted in bold.

For EF, no differences in interpretation of results were observed, whereas, for DP, PF and Burnout prevalence, cross-cultural differences were observed in the interpretation of the results when applying the Gil-Monte criteria against the rest of the cut-off points considered (Table 4).

**Table 4:** Differences in the interpretation of the results of “high degree of affectation of the subscales” and prevalence of Burnout according to the normative criteria considered.

Normative criteria	Emotional fatigue		Depersonalization		Personal fulfillment		Burnout	
	2013	2016	2013	2016	2013	2016	2013	2016
	n (N)	n (N)	n (N)	n (N)	n (N)	n (N)	n (N)	n (N)
	p*	p*	p*	p*	p*	p*	p*	p*
Gil-Monte (Reference category)	36 (64)	40 (61)	32 (68)	17 (84)	41 (59)	16 (85)	14 (86)	5 (96)
Maslach	27 (73) 0.17*	35 (66) 0.467*	11 (89) **< 0.001** *	9 (92) 0.09*	21 (79) **0.002** *	6 (95) **0.02** *	3 (97) **0.005** *	1 (100) 0.09*
Seisdedos	36 (64) 1*	40 (61) 1*	26 (74) 0.35*	16 (85) 0.85*	22 (78) 0.003*	7 (94) 0.04*	7 (93 0.1*	3 (98) 0.47*
Neira	41 (59) 0.46*	45 (56) 0.47*	51 (49) **0.006** *	25 (76) 0.16*	41 (59) 1*	16 (85) 1*	20 (80) 0.25*	5 (96) 1*
Schaufeli	41 (59) 0.46*	45 (56) 0.47*	18 (82) **0.02** *	11 (90) 0.22*	8 (92) **< 0.001** *	3 (98) **0.001** *	3 (97) **0.005** *	1 (100) 0.09*

* Statistical significance of the differences observed in the results of a high degree of affectation of the subscales and Burnout prevalence (Gil-Monte criteria versus the other normative criteria considered) (Chi-square test).

n: Number of individuals with a high degree of affectation of the subscale (3).

N: Number of individuals with medium or low-level affectation of the subscale (1, 2).

Values with statistical significance are highlighted in bold.

The sociodemographic variables showed little association with the outcome variables, whereas a high perception of some of the modulating variables did, however, show an association with Burnout and with the high affectation of the subscales that define it (Table 5).

**Table 5:** Association of sociodemographic, triggering and modulating variables with Burnout subscales according to Gil-Monte cut-off points (2013–2016).

Variable			Emotional fatigue		Depersonalization		Personal fulfillment		Burnout	
			High affectation total; n (%)	Adjusted OR (95%CI)	Alta afectación/total; n (%)	Adjusted O (95%CI)	Burnout/total n (%)	Adjusted OR (95%CI)	Burnout/total n (%)	Adjusted OR (95%CI)
Age (years)	2013	< 50	26/65 (40.0)	2.5^a^	22/65 (33.8)	2.1^a^	28/65 (43.1)	**5.9** a,g	9/65 (13.8)	2.6^a^
		≥ 50	6/25 (24.0)	(0.8–8.1)	6/25 (24)	(0.6–6.9)	6/25 (24.0)	(1.5–23.9)	3/25 (12.0)	(0.4–15.8)
	2016	< 50	26/58 (44.8)	1.7^a^	14/62 (22.6)	7.4^a^	10/60 (16.7)	1.0^a^	5/63 (7.9)	No
		≥ 50	8/25 (32.0)	(0.6–5.2)	1/28 (3.6)	(0.8–66.1)	10/27 (11.1)	(0.2–4.7)	0/28 (0)	valorable
Specific training	2013	No	8/27 (29.6)	0.7^f^	8/27 (29.6)	0.8^f^	14/27 (51.9)	2.1^f^	4/27 (14.8)	1.3^f^
		Si	27/71 (38.0)	(0.3–2.2)	23/71 (32.4)	(0.3–2.4)	26/71 (36.6)	(0.8–6.0)	9/71 (12.7)	(0.3–5.7)
	2016	No	15/34 (44.1)	1.4^f^	7/38 (18.4)	0.5^f^	10/38 (26.3)	**4.5** f,g	3/39 (7.7)	1.5^f^
		Si	21/58 (36.2)	(0.5–4.0)	9/62 (14.5)	(0.1–2.1)	5/59 (8.5)	(1.1–17.9)	2/62 (3.2)	(0.2–13.5)
Appreciated by patients	2013	No	7/14 (50.0)	3.7^f^	7/14 (50.0)	3.2^f^	10/14 (71.4)	**5.3** f,g	4/14 (28.6)	4.2^f^
		Si	22/71 (31.0)	(0.9–15.6)	18/71 (25.4)	(0.8–13.5)	23/71 (32.4)	(1.2–24.8)	6/71 (8.5)	(0.8–22.9)
	2016	No	5/6 (83.3)	8.4^f^	2/7 (28.6)	3.1^f^	4/7 (57.1)	4.4^f^	2/7 (28.6)	15.4^f^
		Si	24/76 (31.6)	(0.9–82.0)	11/81 (13.6)	(0.4–26.1)	10/77 (13.0)	(0.7–27.0)	2/81 (2.5)	(0.9–260)
Appreciated by relatives	2013	No	11/22 (50.0)	**4.2** f,g	10/22 (45.5)	2.9^f^	13/22 (59.1)	3.1^f^	6/22 (27.3)	**4.6** f,g
		Si	18/62 (29.0)	(1.2–14.7)	16/62 (25.8)	(0.9–0.8)	21/62 (33.9)	(0.9–10.5)	5/62 (8.1)	(1.01–21)
	2016	No	8/10 (80.0)	**5.7** f,g	4/11 (36.4)	2.5^f^	5/11 /45.5)	**5.4** f,g	3/11 (27.3)	**24.4** f,g
		Si	20/68 (29.4)	(1.1–32.5)	8/73 (11.0)	(0.4–15.3)	8/70 (11.4)	(1.1-29.0)	1/73 (1.4)	(1.3–450)
Appreciated by superior	2013	No	8/30 (26.7)	0.6^f^	8/30 (26.7)	0.9^f^	13/30 (43.3)	1.4^f^	4/30 (13.3)	1.0^f^
		Si	24/59 (40.7)	(0.2–1.8)	18/59 (30.5)	(0.3–2.7)	22/59 (37.3)	(0.5-3.8)	7/59 (11.9)	(0.2–4.7)
	2016	No	14/25 (56.0)	**3.3** f,g	6/28 (21.4)	1.3^f^	9/29 (31.0)	**9.7** f,g	5/29 (17.2)	No
		Si	18/60 (30.0)	(1.1–10.1)	10/65 (15.4)	(0.3–4.8)	4/61 (6.6)	(1.7–54.3)	0/65 (0)	valorable
Professional experience	2013	↑Perception	11/31 (35.5)	1.1^c^	9/31 (29.0)	1.1^c^	5/31 (16.1)	**0.3** c,g	4/31 (12.9)	1.2^c^
		Rest	24/68 (35.3)	(0.4–2.8)	23/68 (33.8)	(0.4–2.9)	35/68 (51.5)	(0.08–0.8)	10/68 (14.7)	(0.3–4.8)
	2016	↑Perception	5/21 (23.8)	0.6^c^	1/26 (3.8)	0.4^c^	2/26 (7.7)	0.2^c^	0/26 (0)	No
		Rest	31/71 (43.7)	(0.2–2.2)	15/74 (20.3)	(0.04–3.5)	13/71 (18.3)	(0.03–2.1)	5/75 (6.7)	valorable
Optimism	2013	↑Perception	16/45 (35.6)	1.0^e^	13/45 (28.9)	0.7^e^	9/45 (20.0)	**0.2** e,g	5/45 (11.1)	0.6^e^
		Rest	19/54 (35.2)	(0.4–2.5)	19/54 (35.2)	(0.3–1.9)	31/54 (57.4)	(0.07–0.6)	9/54 (16.7)	(0.2–2.2)
	2016	↑Perception	8/31 (25.8)	0.3^e^	6/35 (17.1)	1.6^e^	3/33 (9.1)	0.3^e^	0/35 (0)	No
		Rest	28/61 (45.9)	(0.1–1.0)	10/65 (15.4)	(0.4–5.8)	12/64 (18.8)	(0.07–1.8)	5/66 (7.6)	valorable
Social support	2013	↑Perception	20/63 (31.7)	0.4^e^	19/63 (30.2)	0.9^e^	21/63 (33.3)	**0.3** e,g	7/63 (50.0)	0.4^e^
		Rest	16/37 (43.2)	(0.2–1.1)	13/37 (35.1)	(0.3–2.7)	20/37 (54.1)	(0.1–0.9)	7/37 (18.9)	(0.1–1.6)
	2016	↑Perception	19/59 (32.2)	0.6^e^	8/65 (12.3)	1.1^e^	4/63 (6.3)	**0.07** e,g	1/65 (1.5)	0.2^e^
		Rest	17/33(51.5)	(0.2–1.6)	7/34 (20.6)	(0.3–3.8)	10/33 (30.3)	(0.01–0.4)	3/35 (8.6)	(0.02–2.1)

a Adjusted for gender, time in the job, type of contract and professional category.

b Adjusted for age, time in the job, type of contract and professional category.

c Adjusted for age, gender, time in the job, and professional category.

d Adjusted for age, gender, type of contract and time in the job.

e Adjusted for age, gender, type of contract and professional category.

f Adjusted for age, professional category, type of contract and time in the job.

g p < 0.05

↑ Perception: High perception of the modulating variable considered, considering that it reached this level when its score was higher than 8.

Values with statistical significance are highlighted in bold.

## DISCUSSION

To calculate the prevalence of Burnout, different cut-off points were used, evidencing the importance of selecting appropriate normative values to avoid distortions in the interpretation of the results. The prevalence obtained varied greatly depending on the cut-off points used. The reason for this variability is that, in order to define the cut-off points that evaluate the syndrome, Maslach and Jackson^2^ propose to use the 33rd and 66th percentiles, so that they divide the sample into three equal groups for each subscale. But this method provides different cut-off points depending on the population studied. This variability is due to the occupational and cross-cultural differences of the samples used in the different studies. The cut-off points defined by Maslach for the American population are higher for EF and DP than for Europeans, while their normative values are lower for PF, significant differences were observed in relation to the application of cut-off points obtained in samples of Hispanic language. The most restrictive criterion is that of the Schaufeli because, since it is based on the percentiles obtained on a sample of professionals with occupational stress problems, it is the only criterion validated clinically for the diagnosis of Burnout. In this study, as in other studies^7^ ^,^ ^20^, we obtained lower prevalence when applying this criterion, since these cut-off points only detect clinically relevant cases.

The participation rate was less than 50%, although in similar studies conducted through self-administered and anonymous questionnaires sent to health personnel it is difficult to overcome this rate^7^ ^,^ ^14^. However, some authors had higher rates, above 60%, in studies performed on more specific groups or in the primary care setting^15^. The participation rate increased by 8% in the 2016 cut-off, indicating a certain increase in adherence to the procedure.

There are several studies on burnout and occupational stress in health professionals, but many present methodological limitations. Some studies report low, medium and high percentages of EF, DP, and PF, without considering the means of the subscales nor the prevalence of the syndrome^21^. Others contribute the data of the prevalence of Burnout, but it is difficult to compare the results obtained by different researchers. This difficulty is due to the fact that different normative criteria were used^22^, considering the results on many occasions of American cutoffs^16^ or values of their own tertiles^17^. Some classify as Burnout professionals with a medium or high level of affectation of the three subscales^15^ ^,^ ^18^ or diagnose the syndrome when two of the subscales are affected at high level^23^, oversizing the prevalence of the syndrome. Other studies, such as ours, were performed in small samples or have low response rates, so it is difficult to extrapolate the results^24^. However, almost all studies agree that the main manifestation of the syndrome and the most stable in time is EF^1^ ^,^ ^6^, also observed in our study. Significant differences were observed between the two moments for the DP and PF subscales, but the EF showed no significant changes.

We verified the variability of the percentile statistical criterion proposed by Maslach for the calculation of the population cut-off points since the values obtained in 2016 were significantly different from those of 2013. This variability corroborates the need to establish normative criteria that are standardized, stable and according to the characteristics of the studied population. In MBI, by not defining a clinical criterion of reference that clearly identifies the people who developed the syndrome, the classification is arbitrary and can induce errors in the interpretation of the results, especially when the subscales do not present an adjusted distribution to the normality^20^. Many authors propose using specific cut-offs by country and profession^9^ ^,^ ^12^ ^,^ ^25^ to avoid such distortions. In our study, to research the association between variables, we used the normative criteria defined by Gil-Monte^9^ for a Spanish multi-occupational sample, since they are the ones that best adapt to the characteristics of the studied population.

Analyzing the influence of sociodemographic variables, we observed the association between age and personal achievement in the 2013 cut-off, with greater personal fulfillment in older professionals. These results differ from those observed in other studies, in which the association between certain sociodemographic variables and the presence of Burnout was observed^10^ ^,^ ^15^ ^,^ ^17^ ^,^ ^26^. There is no consensus in the configuration of the epidemiological profile of the syndrome^15^ ^,^ ^18^. Regarding gender, there are studies in which women are more affected^26^ ^,^ ^27^, others find men^11^ ^,^ ^15^ and others do not find significant differences, as was our case.

According to our results, social support acted as a protective factor for PF in both cut-offs, probably because it cushioned work stress, also described in the literature^28^. On the contrary, they acted as risk factors for EF, PF, and the presence of Burnout, low appreciation for patients, family members, and superiors. The DP scale showed no association with any of the variables considered, whereas the PF was the scale that showed a greater number of associations with the explanatory variables, as happened in other studies consulted^10^ ^,^ ^14^ ^,^ ^17^. Lack of training and a negative perception of the assessment by patients, relatives and superiors increased the risk of affectation of the subscale, while a high perception of professional experience, optimism, and social support acted as protective factors.

For some authors, certain personal and work characteristics (chaotic environment, poor interpersonal relations, feelings of inequality) are associated with high levels of Burnout^15^ ^,^ ^29^. Others found an association between Burnout and low levels of empathy^30^ because if the environment is toxic, it changes the dynamics at work and generates stress.

The characteristics of this study do not allow us to draw general conclusions. But it can expose the possibility of reducing professional fatigue by applying measures that reduce the stress in the work and to improve the quality of life of the workers. The efficacy that professionals who need help may have is doubtful^16^. Professional vocation, certain personality traits, and well-rounded teamwork, with clear, realistic goals and assumed by all, are the best antidote to Burnout. Professionals with a positive personality, optimism and self-efficacy are less likely to be exhausted^10^, findings also observed in our study.

Among the limitations of our study is its cross-sectional design, which does not allow conclusions to be drawn by chance or directionality of the relations between the studied variables. Longitudinal studies would be necessary to analyze these relationships. There is a possible distortion of selection since with the participation being voluntary and anonymous, the most proactive professionals are the most participative. The low number of participants is another limitation, given the small size of the organization, which prevents the extrapolation of the results obtained.

Our professionals present an average level of professional fatigue, similar to that obtained in other studies performed in primary care or in chronic care hospitals, although the emotional component affects more our environment.

The prevalence of Burnout varied significantly as a function of the cut-off points applied. This confirms the importance of using normative values according to the characteristics of the studied population to avoid cross-cultural distortions in the interpretation of the results.
